# Substantially thinner internal granular layer and reduced molecular layer surface in the cerebellar cortex of the Tc1 mouse model of down syndrome – a comprehensive morphometric analysis with active staining contrast-enhanced MRI

**DOI:** 10.1016/j.neuroimage.2020.117271

**Published:** 2020-12

**Authors:** Da Ma, Manuel J. Cardoso, Maria A. Zuluaga, Marc Modat, Nick M. Powell, Frances K. Wiseman, Jon O. Cleary, Benjamin Sinclair, Ian F. Harrison, Bernard Siow, Karteek Popuri, Sieun Lee, Joanne A. Matsubara, Marinko V. Sarunic, Mirza Faisal Beg, Victor L.J. Tybulewicz, Elizabeth M.C. Fisher, Mark F. Lythgoe, Sebastien Ourselin

**Affiliations:** a Department of Medical Physics and Biomedical Engineering, University College London, United Kingdom; b Centre for Advanced Biomedical Imaging, University College London, United Kingdom; c School of Engineering Science, Simon Fraser University, Burnaby, Canada; d School of Biomedical Engineering & Imaging Sciences, King's College London, United Kingdom; e Data Science Department, EURECOM, France; f UK Dementia Research Institute at University College London, UK London; g Down Syndrome Consortium (LonDownS), London, United Kingdom; h Department of Radiology, Guy´s and St Thomas’ NHS Foundation Trust, United Kingdom; i Melbourne Brain Centre Imaging Unit, Department of Medicine and Radiology, University of Melbourne, Melbourne, Australia; j The Francis Crick Institute, London, United Kingdom; k Department of Ophthalmology & Visual Science, University of British Columbia, Vancouver, Canada; l Department of Immunology and Inflammation, Imperial College, London, United Kingdom; m Institute of Neurology, University College London, United Kingdom

**Keywords:** Cerebellar cortical laminar structure, Down syndrome, Cortical volume, Cortical thickness, Cortical surface area, Active staining, Contrast-enhanced MRI, Tc1, Cortical morphometric analysis

## Abstract

Down Syndrome is a chromosomal disorder that affects the development of cerebellar cortical lobules. Impaired neurogenesis in the cerebellum varies among different types of neuronal cells and neuronal layers. In this study, we developed an imaging analysis framework that utilizes gadolinium-enhanced *ex vivo* mouse brain MRI. We extracted the middle Purkinje layer of the mouse cerebellar cortex, enabling the estimation of the volume, thickness, and surface area of the entire cerebellar cortex, the internal granular layer, and the molecular layer in the Tc1 mouse model of Down Syndrome. The morphometric analysis of our method revealed that a larger proportion of the cerebellar thinning in this model of Down Syndrome resided in the inner granule cell layer, while a larger proportion of the surface area shrinkage was in the molecular layer.

## Graphical abstract

**Figure:**
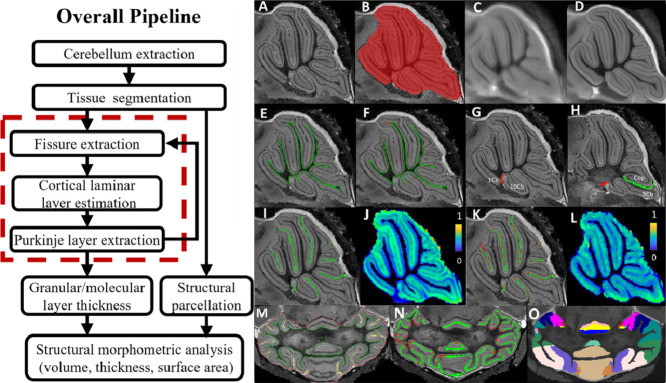

## 1 Introduction

The cerebellum is an important structure in the hindbrain located between the cerebrum and the brain stem. Recent decades of studies have revealed an association between the cerebellum and a diverse range of cognitive functions as well as neuropsychiatric disorders (Schmahmann and Caplan, 2006; Buckner, 2013). Cerebellar damage, deficit or volume change have been shown to correlate with various neurological deficiencies (Schmahmann and Sherman, 1998; Ravizza et al., 2006; Sim et al., 2020; Jung et al., 2019), and particularly with Down Syndrome (DS) (Aylward et al., 1997), one of the most common genetic causes of intellectual disability. DS is caused by the presence of an extra copy (trisomy) of human chromosome 21 (Hsa21) and the resulting overexpression of specific genes that give rise to relevant clinical features (Baxter, 2020; Moldrich et al., 2007; Wiseman et al., 2009; Garc´ıa-Cerro et al., 2020). Having DS is the single biggest risk factor for succumbing to early-onset Alzheimer's disease (AD) (Wiseman et al., 2009; Sheppard et al., 2012; Wiseman et al., 2015; Wiseman et al., 2018).

The morphology of the cerebellum is presented as folia structures separated by different lengths of fissures (Sudarov and Joyner, 2007). The cerebellar cortex consists of three layers: the internal granular layer, the Purkinje layer, and the molecular layer, ordered from white matter towards the extra-axial cerebral spinal fluid (CSF). Each layer is comprised of different cell types corresponding to diverse cognitive and neuronal functions (D'Angelo and De Zeeuw, 2009; Buckner, 2013), and may exhibit different pathologies under different disease conditions (O'Halloran et al., 2012; Wagner et al., 2017). Specifically, the neuronal deficiency in patients with DS may vary among different neuronal cell types across the cerebellar laminae. Studies of 2D histology data with various staining techniques have shown layer-specific pathological changes, such as reduced granule cell and Purkinje cell density (Baxter et al., 2000; Guidi et al., 2011).

It is important to understand the tissue-level morphological changes as a consequence of the cellular-level pathology. Due to the limited resolution of clinical MRI and the highly convoluted nature of the human cerebellar cortex compared to cerebral cortex, studies on cortical layer morphology in neurological disorders tend to focus more on the cerebral cortex (Eickhoff et al., 2007; Wagstyl et al., 2018). Among the studies on the DS cerebellum, majority of them have only analysed gross cortical volume (Sim et al., 2020; Jung et al., 2019). However, more detailed morphometrics such as the thickness and surface area of different cerebellar cortical laminae could provide more insights about neurological disorders and phenotype identification compared to simple volumetric analysis.

Given the genetic similarities between humans and mice, and the anatomical correspondence between the human and mouse brain, the mouse represents a promising model to understand how the cerebellum is affected by neurological disorders (Hermiyanty, 2017). Currently, there is a limited number of studies focusing on quantitative analysis of mouse cerebellar MRI and, as in human studies, all of these studies focus only on volumetric analyses (Ullmann et al., 2012; Szulc et al., 2013; Bergounioux and Delsol, 2013; Steadman et al., 2014).

Using high-resolution *ex vivo* MRI, it is now possible to reveal the different cell layers in the mouse cerebellum. Studies comparing high-field MRI of cortical gray matter with histological staining (Fukunaga et al., 2010; Boretius et al., 2009) showed that the variation of iron and myelin content in different cortical layers produced MR contrasts that reflect the local laminar architectures. Marques et al. (Marques et al., 2010) also demonstrated the ability of T2*-weighted images of the mouse cerebellar cortex to reveal the contrast between the cortical layers - the internal granular layer, the Purkinje layer, and the molecular layer in rats. With the administration of the Gadolinium-diethylenetriamine pentaacetic acid (Gd-DTPA), such interlayer contrast can be further enhanced (Watanabe et al., 2013). The active staining technique - incorporating the gadolinium-chelate MR contrast agent during the fixation process - can achieve high-resolution, high-SNR *ex vivo* structural mouse brain MRI with significantly shorter scanning time due to the reduced *T*_1_ relaxation time (Johnson et al., 2002; Petiet and Johnson, 2010; J.O. Cleary et al., 2011), and produce distinctive contrast between different cellular layers within the mouse cerebellar cortex including the middle Purkinje layer (J.O. Cleary et al., 2011). However, studies of layer-wise morphological variation using 3D MR structural images are lacking. It is thus of great interest to utilize such information revealed in MR to infer detailed morphology in each cerebellar cortical layer.

In this study, we developed an analysis framework to estimate the volume, thickness, and surface area of the cortical laminar layers of the cerebellum in a mouse model of DS (Tc1 (O'Doherty et al., 2005)) using high resolution *ex vivo* MRI data with a gadolinium-induced active staining contrast enhancement technique to achieve high tissue contrast among cortical layers. Our work achieved accurately extraction of the middle Purkinje layer through surface segmentation and, when not visible, extrapolate from the laminar layer model. The extracted Purkinje layer enables us to estimate the structural morphologies - volume, thickness, and surface area - of the two other layers (i.e. the granular, and the molecular layer).

## 2 Methods

### 2.1 Animal experiments and imaging protocols

#### 2.1.1 Ethics statement

This study was conducted following approval by the Ethical Review Process of MRC National Institute of Medical Research and authorization by the UK Home Office. Reporting of all animal experiments complied with the ARRIVE guidelines and were carried out in accordance with the U.K. Animal (Scientific Procedures) Act 1986 under relevant project licence authority. A full implementation and consideration for the 3Rs (http://www.nc3rs.org.uk), where appropriate, was followed in the design and conduct of this work.

#### 2.1.2 Animal model and breeding

The Tc1 mouse model of DS (formal name: Tc(HSA21)1TybEmcf) used in this study (O'Doherty et al., 2005; Gribble et al., 2020; Hall et al., 2016) is a transchromosomic aneuploidy mouse model with an extra copy of Hsa21. Due to the presence of the additional chromosome, the Tc1 mouse line exhibits many phenotypes of human DS, such as motor deficits, mandible malformation, congenital heart defects, short-term memory impairment, synaptic plasticity deficit, and cerebellar neuronal density reduction (O'Doherty et al., 2005; Galante et al., 2009; Dunlevy et al., 2020). Tc1 mice were obtained by breeding Tc1 females to F1 (129S8 x C57BL/6) males. Only male mice were included in the study to control the effect of sex. A total of 28 mice were included in the study: 14 transchromosomic Tc1 mice and 14 wildtype littermate controls.

#### 2.1.3 Animal preparation and ex vivo imaging acquisition protocol

Active stained gadolinium-enhanced *ex vivo* T2* MRI scans of the mouse brains were performed at age 18–21 weeks. Mice were sacrificed with an overdose injection of sodium pentobarbitone. An initial saline flush (1520 ml) was administered to the left ventricle, followed by a perfuse-fixation with 50 ml of 4% buffered formal-saline (Pioneer Research Chemicals, Colchester, UK) with 8 mM Gd-DTPA (Magnevist, Bayer-Schering Pharma, Newbury, UK), both at a flow rate of 3 ml/min. The decapitated intact skulls were then post-fixed in a solution of 4% formal saline and 8 mM Gd-GTPA at 4 °C for 9 weeks.

The *ex vivo* images were acquired following the protocol introduced by Cleary et al. (J.O. Cleary et al., 2011) to optimize the tissue contrast between different neuronal layers. The in-skull brains were imaged on a Varian 9.4T DirectDrive VNMRS system (Varian Inc., Palo Alto CA, USA) with a 26 mm quadrature volume coil (RAPID Biomedical GmbH, Wrzburg, Germany). 3D spoiled gradient echo sequence was used, with the following scanning parameters: *TE* = 4*.*03 *ms, TR* = 17 *ms, FA* = 52*°, FOV* = 20*.*48 × 13*.*04 × 13*.*04*mm*^3^, *matrix* = 512 × 326 × 326, *averages* = 6, *scan time* = 3 h. In addition, we used a multi-brain scanning protocol to image three brains at once to achieve multi-brain separation, orientation correction, and brain mask extraction (Powell et al., 2016).

### 2.2 Image processing framework for mouse cerebellar layer feature extraction

**Fig. 1:**
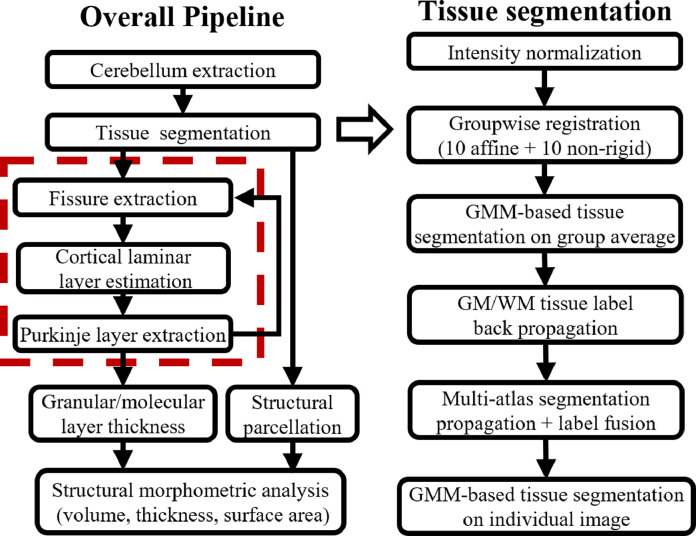
Schematic diagram of the proposed framework. Specifically, the laminar layer separation was achieved through the extraction of the Purkinje layer, as indicated in the steps enclosed by the red square with dashed line.

The overall pipeline of the image processing framework is presented in Fig. 1, which includes the extraction of the cerebellum, the white matter (WM) and gray matter (GM) tissue segmentation, the laminar layer separation, the parcellation of the cerebellar cortex based on the functional characteristics, and finally the measurement of layer-wise morphologies such as volume, thickness, and surface area. Specifically, the laminar layer separation was achieved through the extraction of the Purkinje layer (as indicated in the steps enclosed by the red square with dashed line).

#### 2.2.1 Cerebellum extraction

The cerebellar region was firstly extracted from the original MR images. We adopted the multi-atlas segmentation propagation framework we developed previously (Ma et al., 2014; Ma et al., 2019a) with a publicly available *ex vivo* atlas database created by Ma et al. (Ma et al., 2005) to obtain an accurate structural parcellation. Each atlas template was registered to the target image, first using a symmetric affine registration with block-matching approach (Ourselin et al., 2000; Modat et al., 2014) for global alignment, followed by symmetric non-rigid registration with a cubic B-spline parametrisation of a stationary velocity field (Rueckert et al., 1999; M. Modat et al., 2012; M. Modat et al., 2012) to correct for local misalignment. The corresponding structural labels were then propagated with the resulting deformation field and fused according to the ranking calculated based on the local similarity measurements (Cardoso et al., 2013).

#### 2.2.2 Tissue segmentation

To segment the cerebellar tissue, we first standardized the intensity distribution within the cerebellar area for all MR images. A piece-wise linear intensity normalization (Nyu´l et al., 2000) was used with 11 histogram landmarks defined at [1, 10, 20, ..., 90, 99] percentile. A groupwise average image was then created with an iterative scheme (Powell et al., 2016): one cerebellar image was initially selected at random as the initial reference image, and 10 iterations of affine registration (Ourselin et al., 2000; Modat et al., 2014) were performed to register every other image to the reference image. In each iteration, the affine-registered images were averaged to create a new reference image for the next iteration; another 10 iterations of non-rigid registration (Rueckert et al., 1999; M. Modat et al., 2012; M. Modat et al., 2012) was then performed following a similar iterative strategy.

Tissue segmentation for the GM and WM within the cerebellar mask of the group average image was achieved with a Gaussian mixture model (GMM) of tissue classes (Ashburner and Friston, 2005): 4 tissue types (1 for WM and 3 for GM due to the intensity difference between the cortex laminar layers) were modeled without using anatomical priors and the tissue probability distribution was updated iteratively using an expectation-maximization scheme. Due to the similar intensity between the white matter and the Purkinje layer, the WM segmentation was obtained by firstly manually removing the adjacent misclassified Purkinje layer voxels and keeping the largest connected component. The final GM segmentation was then defined as the cerebellar mask subtracted by the WM segmentation.

The segmented WM and GM were then propagated back to the initial input images using the backward transformations generated during the final iteration of the groupwise registration step. All input images and their back-propagated initial tissue segmentations were then regarded as a new template database to further improve the segmentation accuracy for each image using a leave-one-out multi-atlas segmentation propagation scheme (Chakravarty et al., 2013). A tissue class probability map was generated for each image by first propagating all the initial tissue segmentations from every other image followed by a fusion step with probabilistic output (Ma et al., 2014). A second round of WM/GM tissue segmentations were performed using a GMM, now with the newly generated image-specific spatial anatomical priors.

#### 2.2.3 Initial fissure extraction

Mouse cerebellar cortex is a convoluted folded structure consisting of fissures separating distinctive folia lobules. The adjacent outer surfaces of the lobules touch each other in the fissures, causing partial volume (PV) in MRI and affecting the segmentation accuracy of the CSF that sits inside the fissure. Geodesic-distance-based skeletonization (Han et al., 2004) was used to extract the fissures with the presence of PV. The distance transformation function *D*(*x*) travelled from the white-gray matter boundary surface outwards towards the gray matter-CSF boundary surface was solved with the Eikonal equation F(x)|∇D(x)|=1 where *F*(*x*) = *I*∗ G*_σ_* defines the speed function *F*(*x)* on the Gaussian-smoothed image *I* within the cortical regions, with kernel size *σ* = 1*.*5.

**Fig. 2:**
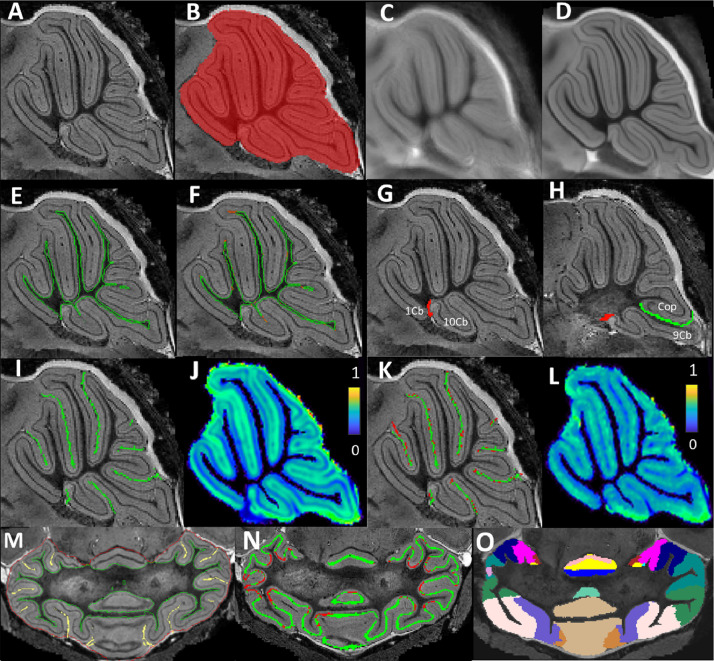
Representative images for each component of the processing pipeline. (A) raw MRI of the cerebellum; (B) cerebellar mask after the cerebellar extracting step; the mean image of the cerebellum across the across the entire studied samples with groupwise (C) affine registration (10 iterations), and (D) non-rigid registration (10 iterations), which exhibit much sharper image contrast compared to the affine-registered average image; (E) WM segmentation after segmentation propagation from the group average image (green), and (F) after applying the leave-one-out segmentation-propagation and label-fusion framework within the testing image group, followed with a further Gaussian mixture model-based tissue segmentation (red); (G,H) The resistant layers were inserted between the 1Cb and 10Cb (red) as well as between 9Cb and 10Cb (green) as boundary conditions to guide the fissure extraction and thickness estimation; (I) Initial fissure extraction based on (J) Speed function calculated from a Gaussian smoothed image intensity (color-coded from 0 to 1); (K) Improved (green) fissure extraction using (L) the speed function after removing the contrast from the Purkinje layer and substitute with the averaged intensity of the surrounding cortical areas(color-coded from 0 to 1); (M) Final gray matter boundaries including: the inner cortical surface touching the white matter (green), the outer cortical surface comprised with the cortical mask boundary (red) and the extracted fissure lines (yellow); (N) Two-step Purkinje layer segmentation, with initial extraction with planar-structure filter modified from the original Frangi's vesselness filter (green), and further improved after applying equivolume-based anatomical laminar model (red). (O) Parcellated cortical structural regions (with each parcellated structured color-coded). (For interpretation of the references to colour in this figure legend, the reader is referred to the web version of this article.)

In the cerebellar vermis, the lobule 1 (1Cb) and lobule 10 (10Cb) touch each other despite being anatomically disconnected. This is also true for the cerebellar vermis lobule 9 (9Cb) and Copula of the pyramis (Cop) in the cerebellar hemispheres (sample illustrations shown in Fig. 2G H in the result section). To prevent these touching lobules from creating false-positive fissure lines when calculating the geodesic distance, resistant layers were added in between with artificially assigned low speed function (close to zero) when calculating geodesic distance for skeletonization. The resistant layers were generated manually from the groupwise average image and propagated back to each individual image. The fissures were then defined as the directional local maxima of *D*(*x*) followed by a skeletonization process using recursive geodesic erosion.

#### 2.2.4 Purkinje layer extraction

After the initial extraction of the fissures, we then extracted the Purkinje layer - a mono-cellular layer consisting of Purkinje cells that sits in the middle of the cerebellar cortex - by exploiting its thin laminar feature. The extraction of the Purkinje layer allowed us to separate and measure the morphological metrics of the molecular and internal granular layers, which sit at the opposite sides of the Purkinje layer.

*a. Planar structure filtering.* The myeloarchitecture within the cortex varies due to the uneven distribution of myelination in different cortical regions, which in turn results in variation of MR intensity and contrast across the cortex (Van Essen and Glasser, 2013; Glasser et al., 2013). Even with the high-resolution postmortem MRI and the active staining contrast-enhanced technique, the extraction of the Purkinje layer is still challenging due to the intensity inhomogeneity and PV effect as well as its thin and highly convoluted nature. Simple thresholding or Gaussian-distribution-based tissue classification methods failed to extract the Purkinje layer without heavy manual intervention. Given the laminar nature of the Purkinje layer, we proposed a planeness filter, which was modified from the Frangi vesselness filter (Frangi et al., 1999; Ma et al., 2015), to find and enhance the image contrast of laminar/planar structures *P(s)* (instead of a tubular structure, as in the original Frangi's vesselness filter formulation) within the cortical region at a fixed scale *s* = 0*.*04:

(1) $$P\left(s\right)\{\begin{array}{c} 0\;if\;\lambda_{2}>0\;or\;\lambda_{3}>0\;\\ exp\left(-\frac{R_{A}^{2}}{2\alpha^{2}}\right)exp\left(-\frac{R_{B}^{2}}{2\beta^{2}}\right)\left(1-exp\left(-\frac{S^{2}}{2\gamma^{2}}\right)\right)\;otherwise \end{array}$$

Where $R_{A}\equiv \frac{|\lambda_{2}|}{|\lambda_{3}|}$, $R_{B}\equiv \frac{\sqrt{\lambda_{1}\lambda_{2}}}{|\lambda_{3}|}$, $S\equiv \sqrt{\sum_{j=1}^{3}\lambda_{j}^{2}}$, and *λ_k_*are the three largest eigenvalue decomposition (|*λ*_1_| ≤ |*λ*_2_| ≤ |*λ*_3_|) of the Hessian Matrix calculated on the Gaussian smoothed image (*σ = 0.5*). Here *R_A_* accounts for the likeness of planar structure as opposed to tubular structure (cross-sectional asymmetry), *R_B_* accounts for the level of deviation from a blob structure which also gives highest response for planar structures, and *S* distinguishes the image structure signal to the background noise (Frangi et al., 1998). *α* = 0*.*5, *β* = 0*.*5 and *γ* = 8 are predetermined thresholds to control the sensitivity of the filter to *R_A_, R_B_* and *S*. The initial estimation of the Purkinje layer $M_{P^{0}}$ was then obtained by taking voxels with strong filter responses. Rigorous quality checks were conducted on the extracted Purkinje layer across all subjects, with remaining false positive filter response manually removed to prevent being propagated to the downstream pipeline.

*b. Cortical laminar layer modeling.* Regions with high curvature cannot be captured entirely using the above planeness filter as the local manifold morphology deviates too much from a planar structure, accompanied by local and contrast inconsistency. These highly curved Purkinje layer regions were then extrapolated from the initial estimation by mathematical modeling of the laminar layer.

The anatomical laminar layer of the cortex was modeled following the Laplacian-equivolumetric model (Leprince et al., 2015). We first reconstructed the pial surfaces by combining the extracted fissures with the outer boundary of cerebellar masks. An initial layer estimation was derived from a Laplacian field level-set analogue between the pial surface and the WM/GM boundary (Jones, 2000). The Laplace equation was solved using the Jacobi method: $\frac{\partial^{2}T}{\partial x^{2}}+\frac{\partial^{2}T}{\partial y^{2}}+\frac{\partial^{2}T}{\partial z^{2}}=0$. The laminar maps were then updated to impose a constant volume across layers (Waehnert et al., 2014). The equivolume-based laminar model was defined along the streamlines of the normalised vector field: $\vec{F}=\frac{\nabla T}{∥\nabla T∥}$. A unit surface *δ_S_*was defined for each cortical voxel on a streamline, and the relative surface area changes of the adjacent points along the streamlines were obtained from the divergence of the vector field $\nabla \cdot \vec{F}:\delta s_{n+1}=\left(1+\delta l\times \nabla \cdot \vec{F}\right)$. An upwind volume towards pial surface *V_pial_*and a downwind volume towards white matter *V_WM_* were calculated by accumulating the relative surface areas of the current voxel *X*_0_ along the streamline towards the points on the pial surface $V_{pial}=\int_{X_{0}}^{X_{pial}}\delta S$. The laminar information on each voxel is defined as the relative volumetric ratios: $R_{vol}=\frac{V_{pial}}{V_{pial}+V_{WM}}$.

*c. Surface extrapolation.* The laminar information obtained from the last step was used to extrapolate the missing parts from the initially extracted Purkinje layers. The Purkinje layer volume ratio was defined at each cortical voxel *R_P_* = *R_vol_* ∗*M_P_*0. A multi-level (= 10) Gaussian smoothing was applied on the volume-ratio-based laminar map *R_vol_* over all voxels $x\in R_{\mathrm{vol}}\cap x\notin M_{P^{0}}$to propagate and average the *R_P_* onto the nearby voxels.

The updated Purkinje layer segmentation extrapolated from the initial estimation was define by finding the directional local minima (*λ* ≈ 0) on the distance map $\lambda =|R_{P_{S}}-R_{WM}|$along the streamline of the vector filed $\hat{V}$. The updated Purkinje layer was then defined as the $M_{P^{F}}=M_{P^{0}}\cup \lambda_{min}$.

#### 2.2.5 Improved fissure extraction with purkinje layer removed

The fissure (sulcal) extraction is an important preprocessing step for cerebellar (cortical) thickness estimation. To account for asymmetric cortical thickness in the sulcal regions, previous studies have used tissue membership functions to guide the skeletonization when extracting deep sulcal lines (Han et al., 2004), which becomes challenging with the existence of the Gd-enhanced Purkinje layer contrast. We have improved the initial geodesic-distance-based fissure line extraction by excluding the “excessive contrast” derived from the Purkinje layer from the speed function for distance calculation and relying solely on the fissure's own contrast when available.

To generate this updated speed map, a multi-level Gaussian smoothing was again applied to the normalized image intensities *I* to all voxels $x\in M_{P^{0}}$to replace the original intensity of the voxels at the Purkinje layer by an averaged value of the intensities in the surrounding cortical voxels. The resulting smoothed intensity images were used as the updated speed function for generating the geodesic distance function to improve the fissure extraction. The laminar modeling and Purkinje layer extraction were then updated accordingly following the updated fissure line.

#### 2.2.6 Cerebellar cortical layer extraction

With the accurate extraction of the white matter, fissure, and the middle Purkinje layer, we then extracted the internal granular layer and molecular layer by utilizing the same laminar-layer-estimation model as described in Section 2.2.4. For the internal granular layer, we first calculated the Laplacian field from the white matter *M_WM_* to the Purkinje layer $M_{P^{F}}$. The resulting volume-preserving Laplacian field region (0<M_G_<1) was then binarized and denoted as the internal granular layer mask. The molecular layer M_M_was defined in a similar way from the pial surface $M_{P^{F}}$to the Purkinje layer $M_{P^{F}}$.

#### 2.2.7 Cerebellar lobule parcellation

Finally, the cerebellar functional lobules were parcellated using a publicly available mouse (C57BL/6 J) cerebellum atlas (Ullmann et al., 2012), which segmented the mouse cerebellum from a groupwise-averaged mean image into functionally distinctive lobular regions. As in step 2.2.2, we used the approach by Chakravarty et al. (Chakravarty et al., 2013) to obtain image specific parcellations based on a leave-one-out segmentation propagation and multi-atlas label-fusion framework. The cerebellar cortex was segmented into a total of 16 cortical lobules as regions of interest (ROI) (Table 1).

**Table 1:** The 16 parcellated cerebellar lobular structures ROI and their corresponding abbreviations. The parcellated labels and the corresponding abbreviation are denoted following the convention as presented in the atlas created by Ullmann et al. (Ullmann et al., 2012).

Abbreviation	Cerebellar cortical structures
*Lobules of the cerebellar vermis*	
1Cb	Lobule 1
2Cb	Lobule 2
3Cb	Lobule 3
4/5Cb	Lobule 4/5
6Cb	Lobule 6
7Cb	Lobule 7
8Cb	Lobule 8
9Cb	Lobule 9
10Cb	Lobule 10
*Lobules of cerebellar hemispheres*	
Sim	Simple lobule
Crus 1	Crus 1 of the ansiform lobule
Crus 2	Crus 2 of the ansiform lobule
PM	Paramedian lobule
Cop	Copula of the pyramis
PFl	Paraflocculus
Fl	Flocculus

### 2.3 Cerebellar cortical layer morphological analysis

To evaluate whether the cortical laminar layer segmentation can provide better insights when comparing the group of Tc1 DS mice with their wild type control littermates, we evaluated and compared the cortical morphologies for the full cerebellar cortex, the internal granular layer, and the molecular layer. Three morphological metrics were measured and compared for each parcellated cortical region: the structural volume, the laminar layer thickness, and the surface area. The relationship between the layer thickness, surface area, and cortical volume were also analysed.

#### 2.3.1 Cortical volume, thickness, and surface area estimation

The thicknesses over the entire cortex *T_GM_*, the internal granular layer *T_Gran_*, and the molecular layer *T_Mol_* were measured at each voxel in the Purkinje layer location $M_{P^{F}}$. The total cortical thickness was defined between the white matter and the pial surface; the internal granular layer thickness was defined between the white matter and the Purkinje layer; and the molecular layer thickness was defined between the pial surface and the Purkinje layer. The voxels in the Purkinje layer were excluded from the thickness measurements. Thicknesses were modeled as the length of the perpendicular streamlines between the two boundaries of each laminar structure of interest and calculated with Eulerian PDE method proposed by Yezzi and Prince (Yezzi and Prince, 2003).

To further analyze the cortical morphology at the structural level, we grouped the morphometrics within each parcellated structure as regions. The average thicknesses for *T_GM_, T_Gran_* and *T_Mol_* for each parcellated cortical region were also calculated. The average surface area in each parcellated region in each laminar layer or the entire cortex was then defined as the structural volume divided by the average regional thickness.

In addition, we also calculated the cortical thickness-to-surface-area ratio (TSR) for each parcellated layer region. The TSR is calculated as $R_{ij}=T_{ij}/\left(\sqrt{V_{ij}}\right)$for each parcellated structure *i* of each animal *j*, in which the ratio *R_ij_* is calculated by dividing the measured thickness *T_ij_* with the square root of the measured surface area *V_ij_*. The square root of the volume is taken to ensure the ratio having a meaningful representation of the proportional change between a 2-dimensional (surface area) and 1-dimensional measurements (thickness). Raw measurement (i.e. without TIV normalization) was used to calculate the ratio to preserve anatomically meaningful measurements.

#### 2.3.2 TIV normalization using normalized residual (W-score)

Before performing groupwise statistical comparison for each morphological metrics of interest - volume, thickness, and surface area, we regressed out any gross effect due to the variation of total intracranial volume (TIV) among subjects using the normalized residual method (Sanfilipo et al., 2004; O'Brien et al., 2006; O'Brien et al., 2011), also known as the W-score (Ma et al., 2018; Ma et al., 2019b; Jack et al., 1997).

To obtain the W-score for each structure of each mouse, a linear regression model was firstly constructed for the normal control group: $M_{i}=\beta_{0}+\beta_{1}TIV_{i}+\varepsilon_{i}$. For each mouse *i*in the normal control group, the morphometric of interest *M_i_* (the cortical volume, layer thickness, or surface area) was firstly modeled as the linear combination of the TIV (denoted as *TIV_i_*) and the residual term (denoted as ɛ_*i*_). The residual term ɛ_*i*_ was then regarded as the normalized morphological metrics for each subject. In order to analyze and the group difference in a standard scale across different measurements (e.g. thickness on a vertex, or volume of a structure). we then calculated the standardized residual $W_{i}=\left(\varepsilon_{i}-\mu_{\varepsilon_{WT}}/\sigma_{\varepsilon_{WT}}\right)$, in which *W_i_*is the w-score defined as the Z-score of the residual term ɛ_*i*_, representing the deviation of the individual's measurements from the reference group's mean $\mu_{\varepsilon_{WT}}$, normalized with respect to the standard deviation of the reference mean:$\;\sigma_{\varepsilon_{WT}}$.

#### 2.3.3 Vertex-wise cortical thickness morphometry

To study the local morphological difference between the control and the transchromosomic group, vertex-wise cortical thickness analysis was performed. The local thickness information that are defined on the extracted Purkinje layer surface of each subject was propagated back to the space of the groupwise template using the inverse deformation field generated during the groupwise registration step. Groupwise comparisons in terms of t-statistics were conducted on each vertex on the group-averaged Purkinje surface. Multiple comparison among the highly correlated adjacent vertices were corrected using the random field theory (RFT) (Chung et al., 2020; Brett et al., 2003).

#### 2.3.4 Structure-based cortical morphometry

Statistical analyses were also performed at the structural level to further analyze the cortical morphology. Four morphological measurements with GLM-based TIV normalization calculated in the previous section was reported - the cortical volume, thickness, surface area, and TSR. The group differences for the metrics of each structure of interest were then determined using an unpaired t-tests between the resulting standard residual of the control and the transchromosomic group. Multiple comparisons were corrected with a false discovery rate set to *q* = 0*.*1.

### 2.4 Data and code availability

The data including the results of this study is available at https://github.com/dancebean/mouse-brain-atlas/tree/master/Tc1_Cerebellum. The results are generated using the NifyReg package (https://github.com/KCL-BMEIS/niftyreg), NiftySeg package (https://github.com/KCL-BMEIS/NiftySeg), the Insight Toolkit (ITK, https://itk.org), as well as the mouse brain multi-atlas-segmentation and morphometric analysis toolkit (MASMAT) (https://github.com/dancebean/multi-atlas-segmentation.

## 3 Results

### 3.1 Mouse cerebellar layer feature extraction

This section shows the results of image processing pipelines to extract the cerebellar layer features for the morphological analysis described in Section 3.2. A sample image from each step of the processing pipeline is illustrated in Fig. 2, and the results are described in detail in the following subsections.

#### 3.1.1 Cerebellar extraction and tissue segmentation

Fig. 2B shows the sample results of an extracted cerebellar mask. After 10 iterations of groupwise affine registration (Fig. 2C), followed by 10 iterations of groupwise non-rigid registration (Fig. 2D), 28 images were averaged into the same stereotaxic location with all the tissue contrast locally aligned. The GMM-based tissue segmentations over the final averaged image were then propagated back to each individual image volume (Fig. 2E), followed by another groupwise leave-one-out segmentation propagation and multi-atlas label-fusion process, which further improved the individual tissue segmentation accuracy especially in the region with narrow gyri (Fig. 2F, green: initial segmentation, red: improved segmentation). It is worth noting that the segmented WM label also contains the cerebellar nuclei.

#### 3.1.2 Fissure and purkinje layer extraction

The resistant layers were inserted as boundary conditions to guide the fissure extraction and thickness estimation (Fig. 2G, H). Initial fissure extraction was then obtained (Fig. 2I) using geodesic distance transformation (Fig. 2J). Fig. 2N illustrates the extraction of the middle Purkinje layer by firstly enhancing the contrast of the planar structure (green), followed by the extrapolation step (red) combining anatomical based laminar model with multi-level Gaussian smoothness, resulting in a much complete representation of the entire Purkinje layer. The final fissure extraction after removing the Purkinje layer is shown in Fig. 2K. Fig. 2M demonstrated the inner cortical surface generated from the white matter segmentation (green), and the outer cortical surface obtained by combining the cortical mask boundary (red) with the extracted fissure lines (yellow).

#### 3.1.3 Cerebellar cortical lobule parcellation

**Fig. 3:**
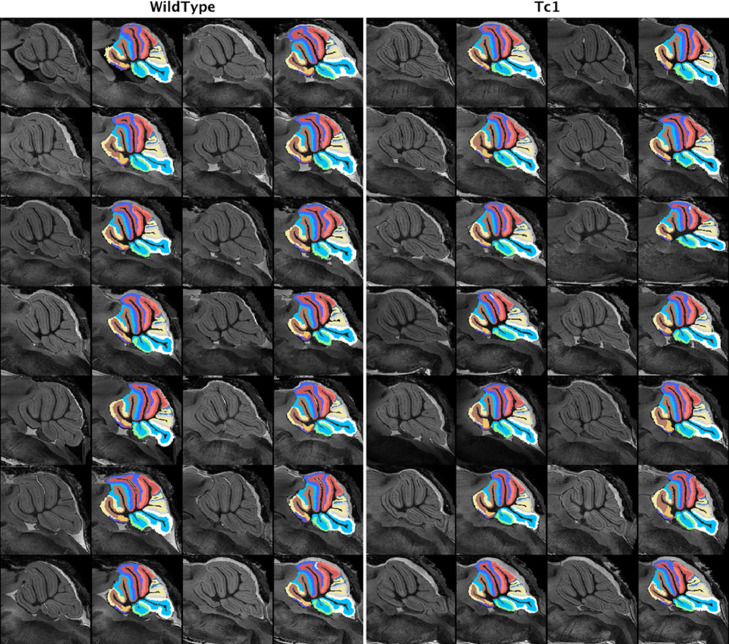
Sample images demonstrate the parcellated cortical lobule structures for both the granular and molecular layers (with each parcellated structured color-coded). Images show the middle sagittal plane of all subjects in the wildtype control and transchromosomic groups. (For interpretation of the references to colour in this figure legend, the reader is referred to the web version of this article.)

The cerebellar cortices are parcellated into different lobular structures, as shown in Fig. 2O. Fig. 3 shows the representation images of the parcellated structural labels on the extracted granular layer and molecular layer in the middle sagittal plane across all the testing subjects. A total of 32 structures (16 in each layer) were segmented in the final parcellated labels, which are used to extract shape-based features for morphometric analysis in Section 3.2.

### 3.2 Cerebellar cortical layer morphological analysis

**Fig. 4:**
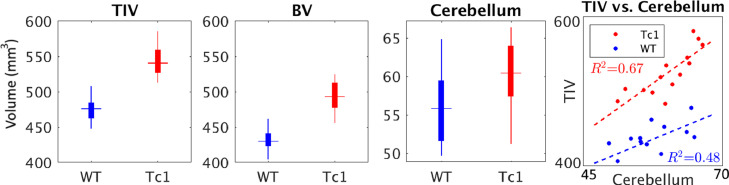
Comparison of the total intracranial volume (TIV), brain volume (BV) and cerebellar volume (Unit: mm^3^). The TIV, BV and cerebellar volume of the Tc1 group are all significantly larger than that of the wild type group.

Fig. 4 shows the of the total intracranial volume (TIV), brain volume (BV) and cerebellar volume in the transchromosomic group and the wild type group. Since a large amount of volume difference is driven by the larger head size, it demonstrates the importance of normalizing the effect of TIV prior to the morphological analyses.

The following sections show the statistical morphometric analysis results in 1) the overall cerebellar cortex; 2) the granular layer; and 3) the molecular layer. Both vertex-wise cortical thickness and structure-based cortical morphometric analysis are reported. All statistical analysis was conducted on the Purkinje layer surface.

#### 3.2.1 Vertex-wise cortical thickness morphometry

**Fig. 5:**
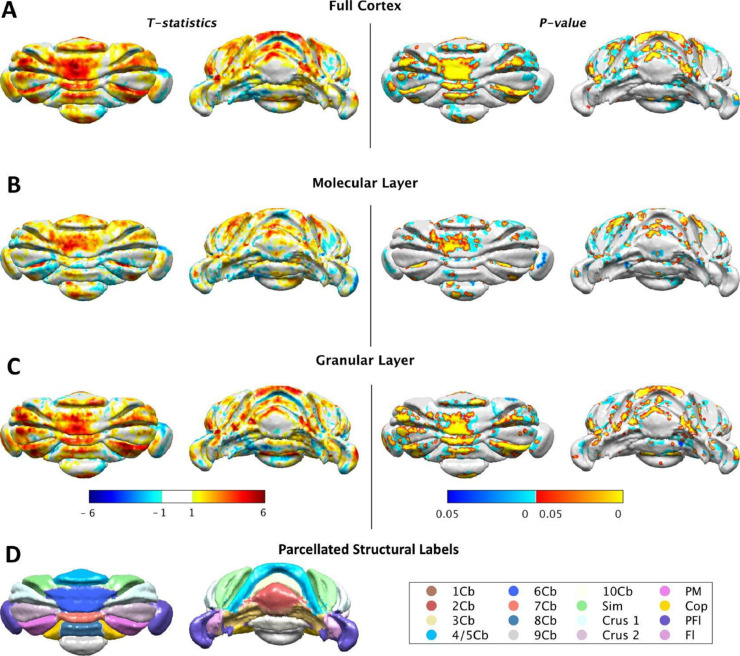
Vertex-wise cortical thickness morphometry performed on the Purkinje layer surface of (A) the full cortex, (B) the molecular layer, and (C) the granular layer. Both the front view (column 1 and 3) and back view (column 2 and 4) of the Purkinje layer are presented. The (left) two columns show the t-statistics, with positive value (red) indicating thicker layer in the wild type group and negative value (blue) indicating the opposite. The (right) two columns show the corresponding significant P-value after controlling the multiple comparison using random field theory (RFT) (Chung et al., 2020; Brett et al., 2003), with blue regions indicate significant clusters, and red regions indicate significant peaks. (D) shows the projected structural parcellation of the cerebellar lobules on the Purkinje layer. (For interpretation of the references to colour in this figure legend, the reader is referred to the web version of this article.)

First, the vertex-wise morphometric analysis demonstrated the localized TIV-normalized cortical thickness difference between the wildtype control and the transchromosomic group. As shown in Fig. 5, the volume-preserving Laplacian thickness of the granular layer (A), the molecular layer (B), and the full cortex (C) were all projected onto the vertexes on the Purkinje layer in the groupwise average space. The corresponding structural parcellation of the cerebellar lobule on the Purkinje layer is also shown in Fig. 5(D) to demonstrate the structural correspondence of each statistically significant regions.

Both the t-statistics (t-map in the left two columns) and the significant P-values (P-map in the right two columns) are presented in Fig. 5. Positive values in the t-statistics (red) indicates where the layer is thicker in the wild type group and negative values (blue) indicate the opposite. Blue regions in the P-map indicate significant cluster regions, while red regions indicate the significant peak regions, as calculated from the RFT. The transchromosomic group showed an overall decrease in the full cortical thickness (C), as well as granular layer thickness (A), with the level of decrease dropping in the molecular layer (B). A symmetric pattern is observed for the thickness difference between the two study groups.

#### 3.2.2 Structural-wise cortical morphometry

**Fig. 6:**
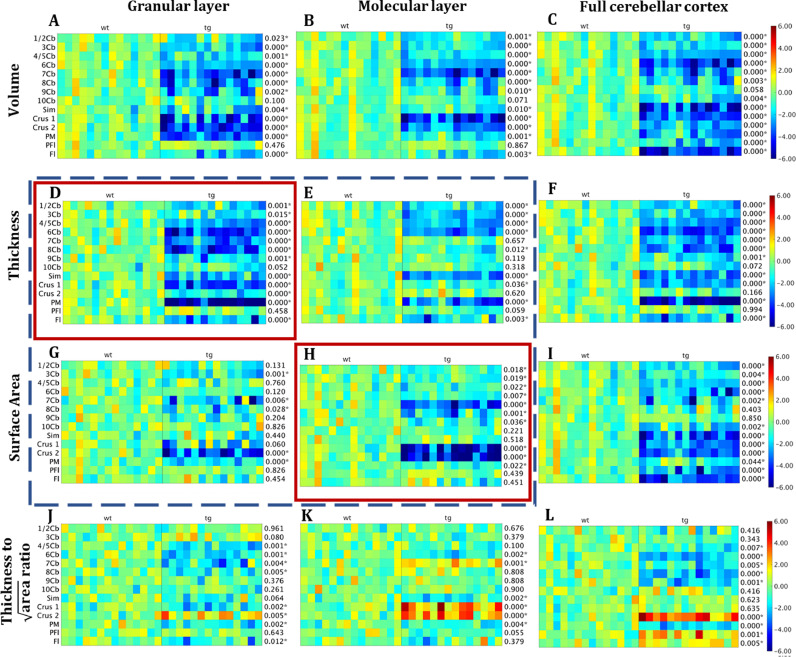
The W-score heatmap shows the panoramic visualization of the W-score of each morphometric measurement on each parcellated structure across all the mouse in both studied groups. The number at the right side of each Zscape subplot shows the P-value of the groupwise statistical comparison results of the *t*-test comparing the Tc1 transchromosomic mouse group with the wildtype littermate using three morphometric measurements - volume (A-C), thickness (D-F), surface area (G-I), as well as thickness to surface area (square rooted) ratio (J-L), measured on the cerebellar internal granular layer (left-column, A,D,G,J), molecular layer (mid-column, B,E,H,K), and full cerebellar cortex (right-column, C,F,I,L). Each row represents a parcellated cortical lobules and each column represent an individual mouse. The linear effect of the TIV has been controlled for all the measurements using the residual-based normalization method, with the residual of the linear fitting as the normalized measurement for comparison. The colors in the figure represent the z-score (standard score) which shows the deviation of the measurements from the mean value of the corresponding reference group (wildtype) and standardized by the reference group standard deviation. The analysis results indicated that most substantial portion of the cerebellar cortical volume reduction (C) comes from the thinning of the internal granular layer (D), followed with the shrinkage of the molecular layer surface area (H). *: Significant difference between the wildtype and the transchromosomic group. (For interpretation of the references to colour in this figure legend, the reader is referred to the web version of this article.)

For each of the parcellated cerebellar cortical lobule, we also analysed three cortical morphology. The lobule 1 and 2 were combined as a single structure due to their anatomical affinity. The linear effect of TIV was controlled using the residual-based normalization methods, W-score, for all measurements. Fig. 6 shows the result of the W-score map, as well as the statistical comparison results (shown as the p-value of the unpaired *t*-test for each structure, presented at the right side of the each W-score map) between the transchromosomic (Tc1) group and the wild type group for all three metrics in each parcellated region. In each subplot, the rows represent parcellated cortical lobules and the columns represent individual mice. Measurements were conducted over: 1) the full cerebellar cortex, 2) the internal granular layer, as well as 3) the molecular layer. In addition, we also calculated the relative change between the raw surface area and thickness, in both the granular and molecular layers, by calculating the ratio between the two. 14 out of the 15 structures were included in the analysis, and Cop was excluded since it sits at the edge of the cerebellar where artificial boundaries were inserted during the processing to avoid the mis-representation of the true underlying anatomical morphologies (as shown in Fig. 2H).

• **Volume** For the full cerebellar cortical volume (Fig. 6C), the transchromosomic group showed significantly smaller normalized cortical volume in 13 out of 14 ROIs (all except 9Cb). For the layer-wise cortical volume, the group differences in the internal granular layer (Fig. 6A) appeared to be more prominent compared to that in the molecular layer (Fig. 6B), although similar levels of statistical significance were observed.
• **Thickness** Cortical thinning happened more prominently in the internal granular layer than the molecular layer. 11 out of 14 segmented cerebellar lobules (all except 10Cb, Crus2, Cop, and PFI) showed significant full cortical thinning in the transchromosomic group (Fig. 6F), while 12 out of 14 structures (with the addition of Crus 2) showed significant internal granular thinning (Fig. 6D). On the contrary, only 9 ROIs exhibited significant molecular layer thinning (Fig. 6E), missing 7Cb, 9Cb, and Crus 2 comparatively.
• **Surface area** Contrary to the result of the cortical thickness, surface area shrinkage appeared to be relatively more prominently in the molecular layer than the internal granular layer. The Tc1 group showed a significantly smaller molecular surface at lobule 1,2,4 and 8 (Fig. 6H) compared to the internal granular layer where no significant difference was found in those regions.
• **Thickness-to-surface-area ratio** The thickness-to- (root-square-of)-surface-area ratio as shown in (Fig. 6J-L) further revealed more subtle connections and interactions between the two morphological measurements and the variation of such distribution among structures. For the full cortex (Fig. 6L), the ratio of Tc1 group was significantly smaller in the central lobules (4–8Cb) and PM, but significantly larger for the other lateral lobules (Crus2, PF1 and F1). For the internal granular layer, the ratio of Tc1 group was significantly smaller in the central lobules (Fig. 6J). For the molecular layer, the ratio of Tc1 was significantly larger in the lateral lobules (Fig. 6K). Interestingly, for structures 7Cb and Crus1, the ratio was significantly smaller for Tc1 group in the internal granular layer (Fig. 6J) while significantly larger in the molecular layer (Fig. 6K). In terms of full cortical thickness, Tc1 group showed significantly smaller ratio in the 7Cb,but no significant difference for Crus1 (Fig. 6L).

When comparing among the three morphological measures, volume analysis showed the most significant overall group difference across all the parcellated cortical structures for both the full cerebellar cortex as well as the internal granular and molecular layers. This is expected as the volumetric difference comprises of both thickness and surface area variations. Interestingly, for the internal granular layer, a relatively large proportion of the volume shrinkage was driven by cortical thinning, while the contribution of the cortical surface area was less significant (Fig. 6A,D,G). On the other hand, the molecular layer exhibited the opposite trend – a greater proportion of the significant volume shrinkage was attributed more to the surface area shrinkage over the layer thinning (Fig. 6B,E,H). Finally, for the full cerebellar cortex, there was a discrepancy between the regions with significantly thinner layers (Fig. 6F) and regions with significantly shrunken surface areas (Fig. 6I) in the Tc1 transchromosomic group, indicating a level of localized morphological variation among the cellular lobules.

In summary, the analysis result revealed that a relatively larger portion of the cerebellar cortical thinning comes from the internal granular layer, while a relatively larger proportion of cortical surface shrinkage occurs in the molecular layer.

## 4 Conclusion & discussion

In this paper, we presented a framework combining the technique of Gadolinium-enhanced active staining *ex vivo* MRI with morphometric analysis to quantitatively analyze the cerebellar cortical laminae of the Tc1 mouse model of DS. Our framework extended the conventional Laplace-equation-based cortical thickness estimation method to laminar layers, and three metrics were measured to quantify the morphometric change in each laminar layer - structural volume, layer thickness, and surface area. The two cerebellar cortical laminae - the internal granular layer and the molecular layer - were segmented through a novel two-step extraction of the middle cellular layer sitting in between them - the Purkinje layer. The proposed method combines the planar filter, modified from the Frangi's vesselness filter, with the laminar-model-based progressive Gaussian filter provide an integrated solution to extract surface structures even with highly curved shape or inconsistent or inhomogeneous intensity profile. We have demonstrated robust performance of the proposed method when applied to extract the Purkinje layer from the mouse cerebellar cortex.

To the best of our knowledge, our results revealed, for the first time in the literature, that the DS-related cerebellar cortical volume reduction involves differentiated morphological changes between the two laminae, with the majority of the cortical thinning coming from the internal granular layer across the whole cerebellar cortex, while surface area shrinkage occurs slightly more in the molecular layer in localized lobules.

The results of this study extend our previous findings (Ma et al., 2014; Powell et al., 2016) using the Tc1 mouse line on the gross shrinkage of the normalized cerebellar volume. There are few existing studies available to validate our findings of the cerebellar layer morphology, especially on the Tc1 mouse. When compared to the previous histological studies using a different mouse model of DS (Ts65Dn) (Baxter et al., 2000; Guidi et al., 2011), our findings agree in the reduced granular cerebellar density. Our study further shows that the large proportion of the cortical thinning occurs in the internal granular layer of the Tc1 mouse. The Ts65Dn mouse model is segmentally trisomic for the distal 12–15 Mb of mouse chromosome 16, while the Tc1 mouse line used in this study is aneuploid and stably transmits a freely segregating copy of Hsa21. A known morphological difference of the two mouse models is that the TIV of Ts65Dn mice is smaller than that of the wild type littermate, while TIV of the Tc1 transchromosomic mice is larger (Powell et al., 2016) (Fig. 4), a gross volume discrepancy that has been taken into consideration with appropriate TIV normalization method (see Section 2.3.2, further discussion on this point were provided in Section 4.3).

### 4.1 Cortical laminar layer modeling and cytoarchitecture

Both cerebral and cerebellar cortices are a laminar structure consisting of layers with different myelination, neuronal cell arrangement and density, as revealed by studies of cytoarchitecture and myeloarchitecture of the cerebral cortex (von Economo et al., 2008; Barazany and Assaf, 2012; Waehnert et al., 2014; Van Essen and Glasser, 2013; Glasser et al., 2013; Shafee et al., 2015; Assaf, 2019) as well as cerebellar cortex (Buckner, 2013; Jung et al., 2019). In order to get a good estimation of the cortical morphologies, such as thickness and surface area, and layer-specific features, such as neuronal cell density, it is necessary to derive a cortical laminar model that follows the actual anatomical arrangement, even with the lack of intrinsic imaging contrast. One of the very first laminar layer models was introduced by Jones et al. using the Laplacian field (Jones, 2000). The equivolume model (Leprince et al., 2015; Waehnert et al., 2014) improves cerebral cortical laminar layer resulted in a better represent the anatomical arrangement (Section 2.2.4 b). When the laminar profile resulting from the original Laplacian field model is compared to the profile after imposing the volume-preserving constraint, the laminae in the latter model are pushed towards the side of surface with lower curvature to preserve equal volumes across adjacent laminae, which aligns better with the anatomical arrangement observed from cerebral cortical cytoarchitecture studies (Waehnert et al., 2014; Leprince et al., 2015).

The anatomically motivated equivolume model for estimating the laminae is based only on the information of cortical surface while ignoring the information of image contrast from within the cortex. As a result, mathematical models alone are still inadequate to capture any variations or irregular shapes within the cortex due to genetic-induced morphological variations or disease-related pathologies. Different cortical layers consist of different cellular types and correspond to distinctive functions. The active stained Gadolinium-enhanced MRI method can provide enhanced exogenous image contrast, for example the Purkinje cell layer within the cerebellar cortex (J.O. Cleary et al., 2011; Watanabe et al., 2013), and thus is able to capture the irregular shape of the internal granular layer and the molecular layer. Our imaging analysis framework managed to extract the layer information from both the enhanced image contrast as well as the prior anatomical knowledge.

Furthermore, many efforts have been made to use endogenous MRI for imaging of the cerebral cortical laminar architecture in vivo or *ex vivo* (Barazany and Assaf, 2012; Trampel et al., 2019). Specifically, studies comparing high-field MRI of cortical gray matter with histological staining (Fukunaga et al., 2010) showed that the variation of iron and myelin content in different cortical layers produces MR contrasts reflecting the local laminar architectures. In addition, it has been shown that multi-parametric MRI can provide parameter maps such as relaxation time, magnetization transfer, or susceptibility (Trampel et al., 2019) maps of the cerebellar tissue. Double-inversion-recovery (IR) MRI, T1W/T2W signal ratio, as well as susceptibility-related contrast (Marques et al., 2010), can also reveal the relative degree of myelination, which has been shown to vary between different regions (Marques et al., 2010; Glasser and Van Essen, 2011). Therefore, investigations on the endogenous MR contrast for in vivo imaging of subcortical laminae, or other layered structures such as hippocampal layers (Watanabe et al., 2013), along with the appropriate analytical tools, would have the potential for clinical translation by providing non-invasive diagnostic tools for neurological diseases associated with cerebral pathologies. However, due to the highly convolved and folding nature of the human cerebellar cortex, visualizing cerebellar layers remains a challenging task, even with the state-of-the-art ex vivo MRI imaging methods at 100-micron resolution (Edlow et al., 2019).

### 4.2 Cortical volumetric, thickness and surface area analysis

Cortical structural abnormalities, such as variations in cortical volume (Popuri et al., 2020), cortical thickness (Rimol et al., 2012; Grand'maison et al., 2013; Lerch et al., 2008; Sawiak et al., 2012) and surface area (Lemaitre et al., 2020; Meyer et al., 2013; Winkler et al., 2010; Winkler et al., 2012), have been shown to correlate with various neurological disorders as well as cognitive functional deficits. Thickness measurement is the most widely used cortical morphological measurement for quantitative analysis of neurodegenerative diseases, both in clinical (Rimol et al., 2012; Grand'maison et al., 2013) and preclinical settings (Lerch et al., 2008; Sawiak et al., 2012). However, previous studies have shown that the cortical surface area and cortical thickness demonstrated different structural properties (Lemaitre et al., 2020; Meyer et al., 2013), and it is important to select the appropriate phenotyping method among cortical volume, surface area and cortical thickness for quantitative analysis (Winkler et al., 2010; Winkler et al., 2012). The morphology of the cortex could vary even when the local volume remains constant. For example, the cortical thickness would decrease if the cortical surface area were to increase. It has also been hypothesised that the thinning of the cortex might be due to the increased volume of white matter as a need to establish a denser connection between different functional regions (primary and secondary auditory regions in this case) (Meyer et al., 2013). Therefore, joint analysis of cortical area and thickness has been suggested as a replacement of the gray matter cortical volume analysis (Winkler et al., 2018).

When comparing the cortical thickness with surface area, previous studies on the cerebral cortex morphology in DS patients have shown increased cortical thickness and reduced surface area (Lee et al., 2020). In this paper, we studied the cerebellar cortex of the mouse, and found that not only does the cerebellar thickness decrease significantly, but the level of cortical thinning is more prominent compared to the surface area shrinkage. In addition, the morphology of the cerebral and cerebellar cortices might be different. Specifically, results of our previous studies (Powell et al., 2016) have demonstrated the local difference between these two structures in the Tc1 mouse model - the enlargement in the cerebrum and the shrinkage in the cerebellum compared to the wild type littermates.

**Fig. 7:**
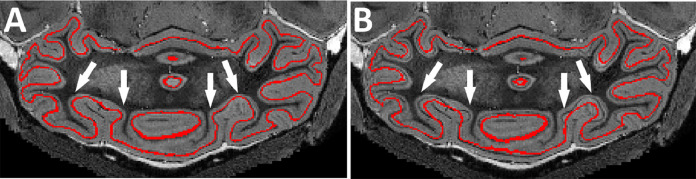
Comparison between the surface representation on (A) the central surface and (B) the Purkinje layer. The white arrow indicated example of regions in the cerebellar cortex where the central surface representation failed to follow the cytoarchitecture, while the Purkinje layer representation does. It can be observed that the projecting the thickness measurement onto the Purkinje layer can provide a more anatomically plausible representation of the cortical morphology.

In order to perform quantitative statistical analyses, many studies on surface-based cortical morphology (most of which use FreeSurfer) represent the cortical thickness/area on the GM/WM surface and/or pial surface (Rimol et al., 2012; Storsve et al., 2014; Meyer et al., 2013). For studies using voxel-based cortical thickness, the voxel-wise thickness maps are commonly projected onto the central surface (Han et al., 2004) (Fig. 7A). The current standard practice of the cortical surface analysis is measurement of the thickness value defined on the mid-cortical surface, which is the half-way between the WM/GM and GM/CSF surfaces (Dahnke et al., 2012). On the other hand, the segmentation of the Purkinje layer provides additional contrast for inter-subject comparison which lead to more robust measurements when performing groupwise statistical analyses (Fig 7B). The Purkinje-layer-based surface representation could potentially provide a more plausible data-driven anatomical-based representation of the cortical surfaces compared to the mid-cortical-surface-based analysis, which is more prominent when studying the layer-wise thickness, volume, and surface thickness.

### 4.3 Anatomical hypothesis for the observed layer-wise morphometry

Anatomically, the internal granular layer consists of densely packed small granule cells receiving signals from the cerebellar nuclei (CN) with their axons (parallel fibers) extending vertically into the outer molecular layer by penetrating the Purkinje layer and then bifurcating into T-shape junctions (Sudarov and Joyner, 2007). This makes the finding of this study - the different morphological alterations between the internal granular layer and molecular layer in the Tc1 mouse model - of great interest. The main findings of the study regarding the Tc1 mouse model can be summarized as follows: 1) A large proportion of the cerebellar volume reduction took place in the internal granular layer; 2) a large proportion of the reduced internal granular layer volume was caused by the layer thinning, while relatively more prominent surface area shrinkage was observed in the molecular layer; 3) the ratio between the thickness and surface area change (TSR) was not distributed evenly across the entire cerebellum, with the central lobules of the internal granular layer tending to show a larger amount of relative thinning, while the lateral lobules of the molecular layer exhibiting greater relative surface area shrinkage. Mechanically, these observations could potentially be explained by that, with a reduced amount of granule cells, a more direct and straightforward effect is cortical thinning, while it is relatively more difficult for the highly folded folia structure surface to shrink unless enough space has been let out due to the granule cell loss. Consequently, a possible reason regarding the second observation could be that the reduced granule cell body in the internal granular layer might potentially induce shrinkage of axonal fibers across both layers, contracting nerve fibers in multiple directions (both perpendicular and parallel to the nerve fiber bundles), causing the molecular layer surface shrinkage as a secondary effect. Finally, for the observation about the different thickness-to-surface-area change ratio between the central and lateral lobule, it might be explained by the fact that the central lobules are anatomically closer to the CN, making them more vulnerable to the above mentioned primary effect of cortical thinning, while on the other hand, the lateral lobules are relative further away from the CN, and therefore more prone to exhibit the secondary effect - surface area shrinkage. We would like to note that it is necessary to conduct further experiments to examine the Tc1 mouse cerebellar at a more microscopic level in order to test the above hypotheses on the underlying mechanism of the results from this study.

### 4.4 The effect and choice of normalization for quantitative morphological analysis

It has been shown that normalization to the TIV for certain morphological analysis is necessary for regressing out gross factors such as normal aging or populational differences (Whitwell et al., 2001; Lerch et al., 2012), and may improve the classification power of quantitative analysis of image studies for diseases such as AD and DS (Westman et al., 2013; Zhou et al., 2014). However, controversies exist regarding which morphological measurements should be regressed out through normalization with TIV, and how the normalization should be performed. Westman et al. have used a FreeSurfer pipeline to investigate the choice of normalization with TIV for different morphological analyses (Westman et al., 2013). Their conclusions support the normalization of volume measurements with TIV, but not the normalization of thickness measurement. On the other hand, another study by Zhou et al. have drawn an opposite conclusion that to obtain the best classification power, the cortical thickness should be normalized with TIV, while such regression is not necessary for volumetric analysis (Zhou et al., 2014).

In this study, we use a linear regression model to perform data normalization (Ma et al., 2019a; Ma et al., 2019b; Harrison et al., 2019), by first modeling the metrics as a linear combination of the TIV with the residual in the wildtype mice and using the W-score, the standardized residual of the model fitting for each individual mice, as the normalised measurement. This residual-based normalization provided a unified framework to take into account any linear factors from the TIV towards any type of morphological measurement - either volumetric, thickness, or surface area. This approach can further be extended to the multiple linear model to regress out any additional covariate in the data that may affect the analysis, such as demographic information like age or sex, as well as device-induced batch effect such as scanner field strength difference (Ma et al., 2019b). W-score is an extension of the Z-score with the incorporate the covariate adjustment and is an effective way to achieve data harmonization (Ma et al., 2019b). W-score has been used extensively for covariate regression while quantifying brain imaging features such as structural volume (Ma et al., 2019a; Popuri et al., 2020; La Joie et al., 2012; Boccardi et al., 2003; Jack et al., 2008). Furthermore, radiomic features standardized with W-score showed improved model performance when trained in deep-learning (D Ma et al., 2020) and other types of machine-learning algorithms (Popuri et al., 2020).

Compared to the division-based TIV normalization (i.e. simply divide the morphometric measurement with the TIV), W-score with residual-based normalization is a more statistically correct and powerful method (Sanfilipo et al., 2004; O'Brien et al., 2006). However, the regression-based normalization requires of sufficient normal control population in the reference group to build a representative normative model and have sufficient statistical power to detect group differences. In this study, we have used the Zscape plotted (Ma et al., 2019b; Popuri et al., 2020; Ma et al., 2017) to show the W-score of each individual morphometric measurements across all subjects, as an addition to the statistical analysis results (Fig. 6), which provide a more informative representation about data distribution across and in-b both groups.

### 4.5 Cerebellar, fissure, and purkinje layer extraction

In this study, multi-atlas label-fusion was used to extract the cerebellar region, which has shown great performance in similar tasks such as brain extraction to calculate the intracranial vault (Ma et al., 2019b). Previously, other studies have tried to extract the cerebellum using pattern recognition techniques such as Mumford-Shah and edge detection (Sijbers et al., 1996; Bergounioux and Delsol, 2013). These methods may rely heavily on image-contrast-specific parameter tuning and may end up including surrounding non-cerebellar tissues and involve heavy user intervention and correction for post-processing. In addition, machine learning methods have also been shown to provide improved accuracy for cerebellar segmentation during label fusion step (Powell et al., 2008). Finally, among the handful of available atlases providing mouse cerebellar lobule parcellations (Ullmann et al., 2012; MacKenzie-Graham et al., 2004; Johnson et al., 2010; Hawrylycz et al., 2014), there is usually only a single average template provided, inducing an extra layer of difficulty for multi-atlas label fusion, and different cortical layers are not distinct. In this study, with the combination of groupwise registration and multi-atlas label propagation and fusion technique, we effectively build a comprehensive mouse cerebellar segmentation database with distinctive lobular structures for each cortical layer.

Within the image processing pipeline, the fissure lines were extracted as the local maxima of the geodesic Euclidean distance map from the WM/GM surface with the travelling speed defined as the smoothed image intensity. With the original image intensity, the speed variations are induced by both the contrast at the Purkinje layer as well as the fissure line when available, introducing local misalignment (Fig. 2K red). After the intensity at the Purkinje layer location was substituted by the smoothed average intensity of the surrounding cortical area, the speed variation is dominated only by the contrast from the fissure line (Fig. 2K green), therefore correcting some local misalignment in the initial fissure extraction. Most improvements were localised at highly curved gray-matter/pial-surface boundary where fissure line ends, correcting the local morphological estimations such as cortical thickness and surface area. However, it is worth noting that the improvement depends highly on accurate Purkinje layer detection. Any missing parts would induce further inaccuracy even in the improved version of fissure extraction, which indicate the importance of proper quality control.

Finally, in the step of extracting the Purkinje layer, when the initial step did not achieve full detection, the combination of equivolume model and progressive Gaussian smoothness was used to recover the Purkinje layer to its entirely. Although this represents the best efforts available to recover the underlying anatomical features, there are still chances of underestimating the curvature feature of the Purkinje layer in the highly curved regions. This effect would be prominent for vertex-wise analysis and less prominent in the structure-based analysis where local morphological changes are averaged across each structure. Additional quality control and manual correction may be needed to achieve the most accurate morphological representation in such areas where the Purkinje layer are recovered by model-based methods rather than intensity-based.

### 4.6 Limitation of the current study and potential future works

In this study, we presented a detailed morphometric analysis of the mouse cerebellar cortex using the enhanced cortical layer contrast with the active staining technique. Compared to histology studies, *ex vivo µ*MRI is advantageous in terms of preserving the tissue integrity and morphology. On the other hand, the postmortem process during the ex-vivo perfusion procedure still would potentially limit the ability of the morphometric analysis to represent the actual underlying tissue composition in the live animal. Ideally, these limitations could be resolved by in vivo imaging. However, due to the constraints such as the relatively short scanning times, animal motion artefacts, and limited methods for inducing imaging contrast, the current state-of-the-art in vivo imaging methods are still unable to provide enough image resolution and contrast for conducting the type of morphological analysis presented in this study (Holmes et al., 2017; Lerch et al., 2012; Ma et al., 2019a). Some studies have managed to achieve in vivo imaging with satisfactory in-plane resolution and contrast to reveal the cytoarchitecture and myeloarchitecture of distinct gray matter layers (Watanabe et al., 2013; Boretius et al., 2009). However, this requires the off-plane slice thickness to be 10 times thicker, thus hindering the feasibility to perform accurate 3D morphological analysis. Therefore, a combination of in vivo and *ex vivo* imaging would be required to perform a thorough morphological study, especially in the case of longitudinal studies, as described in more detail in our previous study (Ma et al., 2019a).

In addition, the underlying connection between the thinner granular cortex and smaller molecular surface area provide an interesting insights to further study the underlying anatomical and physiological mechanisms, although the current resolution and contrast provided by MRI is not sufficient to explore the relationship between the internal granular layer thinning and the molecular layer surface shrinkage at the physical/geometric level, even with the help of active-staining technique. It would be interesting to further investigate these in future studies involving imaging techniques such as histology, ex vivo diffusion tensor MRI, or light sheet microscopy.

Furthermore, in this study we only looked at the morphological changes in the cerebellar laminar layers such as the structural volume, thickness, and surface area. However, the morphological changes in the mouse model do not always represent the corresponding cognitive changes (Gutierrez-castellanos et al., 2013). Therefore, it would be interesting to study and build a more in-depth understanding of the structural-functional correlation in the cerebellum, which would provide important insights to study disease pathology in animal models, and provide translatable conclusions for the development of potential early treatment or prevention strategies.

Finally, in this study, we did not explicitly segment the cerebellar nuclei (CN) which is a deep gray matter structure inside the cerebellar. However, a recent study has revealed the important role of the CN in regulating the developmental scaling of cerebellar cortical cell numbers (Willett et al., 2019). To further understand the source of the changes in the cerebellar cortex, it would be interesting to extend the current work to segment the CN and quantify its connection with the cerebellar cortical layers.

## Credit author statement

**Da Ma:** Conceptualization, Investigation, Methodology, Software, Data curation, Formal analysis, Visualization, Writing - Original Draft, Review & Editing

**Manuel J. Cardoso:** Conceptualization, Investigation, Methodology, Software, Validation, Writing - Review & Editing, Supervision

**Maria A. Zuluaga:** Methodology, Software, Investigation, Writing - Review & Editing

**Marc Modat:** Conceptualization, Methodology, Software, Writing - Review & Editing

**Nick M. Powell:** Methodology, Software

**Frances K. Wiseman:** Investigation, Resource, Writing - Review & Editing

**Jon O. Cleary:** Investigation, Resource, Data curation, Writing - Review & Editing

**Benjamin Sinclair**: Methodology, Validation, Data curation

**Ian F. Harrison:** Validation, Project administration, Writing - Review & Editing

**Bernard Siow:** Project administration, Validation

**Karteek Popuri:** Visualization, Validation

**Sieun Lee:** Validation, Visualization, Writing - Review & Editing

**Joanne A. Matsubara**: Validation, Writing - Review & Editing

**Marinko V. Sarunic**: Validation, Supervision

**Mirza Faisal Beg:** Validation, Supervision

**Victor L.J. Tybulewicz:** Methodology, Investigation, Resource, Writing - Review & Editing, Funding acquisition

**Elizabeth M.C. Fisher:** Methodology, Investigation, Resource, Writing - Review & Editing, Supervision, Funding acquisition

**Mark F. Lythgoe:** Methodology, Investigation, Resource, Writing - Review & Editing, Funding acquisition, Project administration, Supervision

**Sebastien Ourselin:** Methodology, Investigation, Resource, Writing - Review & Editing, Funding acquisition, Project administration, Supervision
